# Efficacy of Oseltamivir Against Seasonal Influenza H1N1 and the Efficacy of a Novel Combination Treatment In Vitro and In Vivo in Mouse Studies

**DOI:** 10.1111/irv.70176

**Published:** 2025-10-20

**Authors:** Danlei Liu, Ka‐Yi Leung, Ruiqi Zhang, Hoi‐Yan Lam, Yujing Fan, Xiaochun Xie, Wan‐Mui Chan, Kelvin Kai‐Wang To, Kwok‐Hung Chan, Ivan Fan‐Ngai Hung

**Affiliations:** ^1^ Department of Medicine Li Ka Shing Faculty of Medicine, University of Hong Kong Hong Kong China; ^2^ Department of Microbiology Li Ka Shing Faculty of Medicine, University of Hong Kong Hong Kong China; ^3^ State Key Laboratory for Emerging Infectious Diseases Li Ka Shing Faculty of Medicine, University of Hong Kong Hong Kong China; ^4^ Carol Yu Centre for Infection Li Ka Shing Faculty of Medicine, University of Hong Kong Hong Kong China; ^5^ Centre for Virology, Vaccinology and Therapeutics Hong Kong Science and Technology Park Hong Kong China

**Keywords:** antiviral treatment, hemagglutinin, influenza, neuraminidase

## Abstract

**Background:**

Influenza surveillance and drug resistance testing have always been central to clinical efforts. Therefore, researching the virus characteristics and antiviral drugs is essential.

**Method:**

The HA and NA activities were assessed in influenza strains, and mutations were identified through gene sequencing. The effects of oseltamivir, molnupiravir, and baloxavir treatments were evaluated in vitro. The effectiveness of molnupiravir monotherapy and its combination with baloxavir was also evaluated in a mouse model. Changes in body weight and lung tissue were examined, including pathological changes, virus replication, and inflammation levels.

**Results:**

Forty‐one seasonal influenza H1N1 strains from 2023 were used. The EC_50_ of oseltamivir was significantly increased compared to the 2009 reference strain. Correlation analysis showed that the increase in EC_50_ was related to the HA and NA activities. The antiviral effects of molnupiravir and baloxavir significantly inhibited virus replication; the combination treatment of molnupiravir/baloxavir showed more potent and synergistic inhibitory effects in vitro. In the mouse model, molnupiravir treatment effectively inhibited virus replication and lung inflammation, but the treatment did not improve weight loss or reduce mortality. With the molnupiravir/baloxavir treatment, viral replication was significantly inhibited and proved to be more effective than either monotherapy. The combination therapy also showed the lowest inflammatory response along with a higher survival rate.

**Conclusions:**

The increase in HA and NA activities of seasonal influenza reduced the efficacy of oseltamivir treatment, but the effectiveness of molnupiravir and baloxavir was retained. Combination therapy showed a significant antiviral effect, which provides a reference for the clinical treatment.

## 1 Introduction

Influenza, a causative agent of seasonal respiratory infectious diseases, poses a threat to human health and lives every year. Especially after the COVID‐19 epidemic, the removal of infection control measures has accelerated the resurgence of influenza. Active and early antiviral intervention can help control the infection rate and reduce hospitalization and mortality among high‐risk groups.

Influenza A viruses (IAVs), one of the common influenza types, are characterized by their subtypes of surface glycoproteins, hemagglutinin (HA), and neuraminidase (NA) [1]. IAV replication begins with the binding of HA to sialylated cell surface receptors, leading to the entry of the virion into the cell via the endosome [2]. The HA intermediate fuses the viral and endosomal membranes, releasing the genome into the cytosol [3]. The viral ribonucleoprotein (vRNP) bundle enters the nucleus through the classical importin pathway, initiating viral mRNA synthesis [4] and the replication of viral genomic RNA [3]. Viral RNA is transported from the nucleus, new viral proteins are synthesized in the cytoplasm, and viral components are assembled at the plasma membrane, initiating budding from the cell membrane. The nascent virion is released from the sialylated cell surface through the sialidase activity of NA [3, 5]. Seasonal influenza is susceptible to point mutations, resulting in significant changes in antigenic sites, leading to antigenic drift and, consequently, the emergence of new influenza virus subtypes that evade the immune pressure of the population [6]. Mutations in influenza may lead to the emergence of a new strain pandemic, render antiviral treatment less effective, and cause drug resistance.

Currently, antiviral drugs such as neuraminidase inhibitors (NAIs) and cap‐dependent endonuclease inhibitors (CENIs) are mainly used in clinical settings against influenza [7]. NAIs, such as oseltamivir and zanamivir, work by inhibiting the activity of NA on the virus surface, thereby preventing the release of influenza viruses [8]. However, NAI susceptibility is monitored annually due to concerns about its potential decline from ongoing mutations in neuraminidase [9]. CENIs represent a new class of anti‐influenza drugs, such as baloxavir marboxil (BXM), which has received approval for clinical use [7]. BXM is known to inhibit vRNA synthesis by blocking the polymerase acidic protein (PA) endonuclease during the cap‐snatching process [7, 10]. However, mutations in seasonal influenza may lead to increasing resistance to current antiviral drugs.

Recent influenza strains require monitoring and assessment of their characteristics and drug resistance. Accordingly, clinical samples from confirmed patients were collected, and influenza strains were isolated in our study. This study aims to observe the characteristics of seasonal influenza and explore suitable new treatment options both in vitro and in vivo.

## 2 Materials and Methods

### 2.1 Viruses, Cell Lines, and Compounds

The seasonal influenza strains were isolated from patients diagnosed with influenza A/H1N1 in Hong Kong in March 2023. The reference H1N1 strain A/Hong Kong/415742/09 was used in vitro, and the A/Hong Kong/415742/09 mouse‐adapted H1N1 strain was used in a mouse model. Virus stock was prepared and titrated by the median tissue culture infectious dose (TCID_50_) on Madin‐Darby canine kidney cells (MDCK). Oseltamivir acid (Oseltamivir carboxylate, MedChemExpress, USA) was dissolved in water (100 mM), while molnupiravir (EIDD‐2801, MedChemExpress, USA) and baloxavir (baloxavir acid, MedChemExpress, USA) were dissolved in DMSO (50 and 10 mM) for in vitro studies. Additionally, molnupiravir and BXM (MedChemExpress, USA) were dissolved in 50% PEG300 in water for animal treatments. All the compounds were stored at −80°C.

### 2.2 Cell Viability Assay

The virus was inoculated onto MDCK cells and incubated at 37°C for 1 h. Then, the wells were washed once, and the drugs were replaced with serial concentrations and incubated at 37°C again. After 48 h, the supernatant was discarded, and 0.5 mg/mL 100 μL of Thiazolyl Blue Tetrazolium Bromide (MTT) was added into each well and incubated at 37°C for 3 h in the dark. Then 100 μL of 0.01‐M SDS‐HCl was added to dissolve the precipitated formazan crystals. Absorbance was measured at 560 nm, and cell viability was calculated as OD (treated–untreated) divided by OD (cell control–untreated). The 50% effective concentration (EC_50_) was further calculated based on cell viability.

### 2.3 Virus, HA/NA Titer and Quantification

The viral titer was determined using the TCID_50_ method. The HA assay was performed on V‐bottomed 96‐well plates to determine HA titer by mixing the twofold diluted virus with 0.5% turkey blood, determining the virus titer by observing the sedimentation of red blood cells after 30 min. The NA titer and NA inhibition by oseltamivir were tested using the NA‐Fluor influenza neuraminidase assay kit (Applied Biosystems, USA), and the procedures followed the product instructions. Quantification was performed using RT‐qPCR. The viral RNA was extracted using viral DNA/RNA extraction kits (Tianlong, China) on the Tianlong instrument. RT‐qPCR for Influenza H1N1 detection was conducted with the QuantiNova Probe RT‐PCR kit (Qiagen, Germany) on the StepOne Real‐Time PCR system (Applied Biosystems, USA). The primers used for detection were designed to target influenza M, H1, and N1 genes, with the sequences shown in Table S1.

### 2.4 Influenza Gene Sequencing and Analysis

Influenza HA and NA gene sequencing was performed using the Oxford Nanopore MinION device (Oxford Nanopore Technologies, UK). The sequence results were analyzed using GISAID FluSurver. The details refer to a previous study [11].

### 2.5 Antiviral Treatments In Vitro

All strain titers used in both mono‐ and combination treatments were diluted to 0.01 MOI. In the monotherapy, the dose range of oseltamivir was from 0.20 to 800 μM. The dose ranges for molnupiravir and baloxavir were from 1.56 to 200 μM and from 0.002 to 0.25 μM, respectively. All the drug concentrations showed no apparent toxicity (Table S2). The drugs were diluted two‐fold for cell treatment, and the EC_50_ was evaluated after 48 h. In the molnupiravir/baloxavir combination treatment, the concentration of molnupiravir ranged from 6.25 to 100 μM, whereas baloxavir ranged from 0.0039 to 0.0625 μM. Cell viability was determined after 48 h, and the results were analyzed using Synergy Finder to identify the concentration range exhibiting a synergistic effect. The supernatants were collected after 48 h and tested for viral titer by TCID_50_ and viral load by RT‐qPCR. The primers used for detection were designed to target the influenza M gene (Table S1).

### 2.6 Antiviral Treatments In Vivo

Female 6–8‐weeks old BALB/c mice were provided by the Center for Comparative Medicine Research (CCMR) at HKU. The A/Hong Kong/415742/09 mouse‐adapted H1N1 strain was used. Seasonal influenza infections may not be consistently susceptible in the mouse model and were, therefore, not included in our in vivo study. Molnupiravir and baloxavir showed similar efficacy against the A/Hong Kong/415742/09 and seasonal influenza strains in our in vitro study, so only the A/Hong Kong/415742/09 mouse‐adapted H1N1 strain was used in the animal model. Mice were intranasally inoculated with H1N1 at 2 × 10^3^ TCID_50_ (10 times the 50% mouse median lethal dose, 10MLD_50_) per 20 μL after being anesthetized; the aim was to evaluate the efficacy of antiviral treatments in moderate to severe infections. This strain has been used and validated in previous studies [12]. Mice were weighed daily to monitor the body weight changes, and any animal showing over 20% body weight loss or signs of severe disease was euthanized. In the molnupiravir monotherapy, mice were divided into three groups: high dose (250 mg/kg per dose), low dose (100 mg/kg per dose), and placebo group. The doses used were safe for mice and were lower than the teratogenic dose for pregnancy [13]. The experimental procedure was shown in Figure 3a.

According to the results of the molnupiravir monotherapy, molnupiravir (250 mg/kg) was selected for combination treatment. The dosage and frequency of BXM were based on a previous study, which showed that plasma drug concentrations were considered close to a human single dose when mice were treated with 15‐mg/kg BXM twice daily [14]. In the combination treatment, a molnupiravir (250 mg/kg)/BXM (15 mg/kg) combo group, two monotherapy groups, and a placebo group were established. The treatment was divided into 3‐ and 5‐day treatments. The survival rate was monitored for 14‐day postinfection (six mice per group). The experimental procedures were shown in Figure 5a,b. The sample size was calculated using the Power and Animal Number Calculator provided by the Committee on the Use of Live Animals in Teaching and Research at HKU.

### 2.7 Histopathologic, Immunologic, and Virologic Assays for Mouse Lung Tissue

After sampling at 4 dpi (six mice per group), the left lung tissues were fixed in 10% formalin and embedded in paraffin. The sections were stained with hematoxylin and eosin (H&E) for light microscopic examination. Based on a previous study [15], a semiquantitative scoring system was used to assess lung lesions. Damage was judged by the infiltration and exudation in the peribronchiolar, perivascular, and alveolar spaces, as well as luminal cell detachment. Scores were 0 = no lesion, 1 = lesion area up to 5%, 2 = up to 25%, 3 = up to 50%, and 4 = more than 50%. The right lung tissues were homogenized for virologic testing. The virus titer was assessed by plaque assay on the MDCK cell line, and the RNA expression was determined by RT‐qPCR. The RNeasy Mini Kit (Qiagen) was used to extract total RNA. PrimeScript RT‐Master Mix (TaKaRa) was used to reverse‐transcribe RNA to cDNA. RT‐qPCR was performed on the StepOne Real‐Time PCR system (Applied Biosystems) using the SYBR Premix Ex Taq II kit (TaKaRa). Primers were used to detect the influenza H1N1 M gene and cytokine/chemokine genes (Table S1). The β‐actin gene served as a housekeeping gene to normalize gene expression, and relative quantification was calculated by using the 2^‐ΔΔct^ method.

### 2.8 Statistical Analysis

The data were analyzed using GraphPad Prism software. Nonlinear regression was employed to determine the EC_50_ and IC_50_. Spearman correlation analysis assessed the relationship between drug effects and influenza HA/NA. The Student's *t*‐test and one‐way ANOVA were used to compare viral replication and cytokine expression among the groups. The Kolmogorov–Smirnov test was conducted to evaluate normality, while the Mann–Whitney and Kruskal–Wallis tests were applied to non‐normally distributed data. The Log‐rank Mantel–Cox test was used to analyze the survival rate in the animal study. A *p*‐value < 0.05 was considered statistically significant.

## 3 Results

### 3.1 Efficacy of Oseltamivir Treatment Against Seasonal H1N1 Influenza In Vitro

**FIGURE 1:**
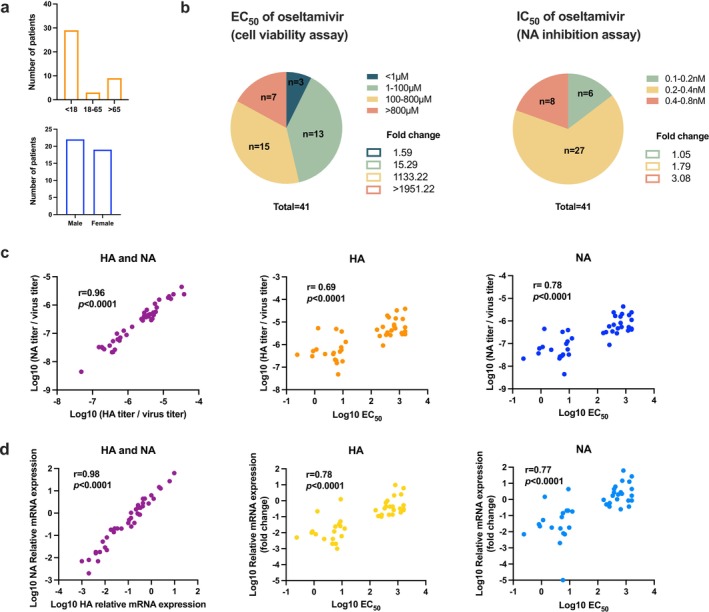
Seasonal influenza activity and its correlation with the effects of oseltamivir treatment. (a) Age and gender distribution. (b) The fold change compared to A/Hong Kong/415742/09 (EC_50_ = 0.41 μM, IC_50_ = 0.16 nM). (c) The correlation between HA/NA activity and EC_50_ of oseltamivir. (d) The correlation between HA/NA quantification and EC_50_ of oseltamivir, as well as the fold change in mRNA expression compared to the reference H1N1 (A/Hong Kong/415742/09), was calculated using the 2^‐Δct^ method, ΔCt = Ct (seasonal strain)—Ct (reference strain). All strains were diluted to an equal virus titer before RNA extraction and RT‐PCR.

Forty‐one H1N1 strains collected from patients in Hong Kong in March 2023 were isolated, primarily from individuals under 18‐ and over 65‐years old, with no significant difference in gender distribution (Figure 1a). The EC_50_ (cell viability assay) of the 2009 reference strain was 0.41 μM, and 3 of the 41 strains exhibited similar EC_50_ values. Among the 41 seasonal strains, the EC_50_ values were below 100 μM for 16 strains, between 100 and 800 μM for 15 strains, and exceeded 800 μM for 7 strains (Figure 1b). However, the NA inhibition capability of oseltamivir was not significantly decreased in the NA inhibition assay. According to the WHO‐AVWG criteria, all strains exhibited normal inhibition by oseltamivir with less than a ten‐fold change in IC_50_. The IC_50_ of oseltamivir against all strains ranged from 0.1 to 0.8 nM, and the IC_50_ of the reference strain was 0.16 nM (Figure 1b). The results demonstrated that there were no major NA mutations in these strains. The correlation between HA and NA activity was highly significant (*r* = 0.96, *p* < 0.0001) (Figure 1c). It also indicated that the EC_50_ had a high correlation with both HA and NA activities (*r* = 0.69 and *r* = 0.78, *p* < 0.0001) (Figure 1c). Furthermore, there was a high and significant correlation between HA and NA quantification (*r* = 0.98, *p* < 0.0001), and the correlation between EC_50_ and the quantification of both HA and NA was significant (*r* = 0.78 and *r* = 0.77, *p* < 0.0001) (Figure 1d). Gene sequencing was performed to investigate the seasonal influenza HA and NA mutations in 7 highly active strains (EC_50_ > 800 μM). All 7 strains belonged to clade 6B.1A.5a.2a and exhibited no major mutations in HA and NA. However, the FluSurver report indicated that these strains exhibited mutations related to mild drug resistance compared to A/California/07/2009/H1N1 [16, 17], and the shift in host specificity and HA‐mediated membrane fusion has increased over the years [18, 19, 20, 21] (Table 1).

**TABLE 1:** NA/HA sequencing results for strains with high viral activity.

Strain no.	2021^**†**^	2019^**‡**^	2009^**§**^
NA			
1	—	—	I188T, V264I, N248D, I314M, I389K, K432E
2	—	—	I188T, N248D, V264I, I314M, I389K
3	—	—	I188T, N248D, V264I, I314M, I389K
4	K331R	K331R	I188T, V264I, N248D, I314M, I389K, K432E, K331R
5	—	—	I188T, N248D, V264I, I314M, I389K
6	—	—	I188T, N248D, V264I, I314M, I389K
7	—	—	I188T, N248D, V264I, I314M, I389K
HA			
1–7	N111D	N173K, V204D	N173K, S200P, Q206E

*Note:* The gene sequence analysis was performed using GISAID FluSurve. Mutations compared to ^**†**^H1N1 A/Sydney/5/2021, ^**‡**^A/Guangdong‐Maonan/SWL1536/2019 and ^**§**^A/California/07/2009. The listed NA mutations were linked to drug sensitivity, and the HA mutations were associated with a shift in host specificity and may enhance HA‐mediated membrane fusion.

### 3.2 Efficacy of Molnupiravir and Baloxavir Treatments Against the Influenza A H1N1 Virus In Vitro

**FIGURE 2:**
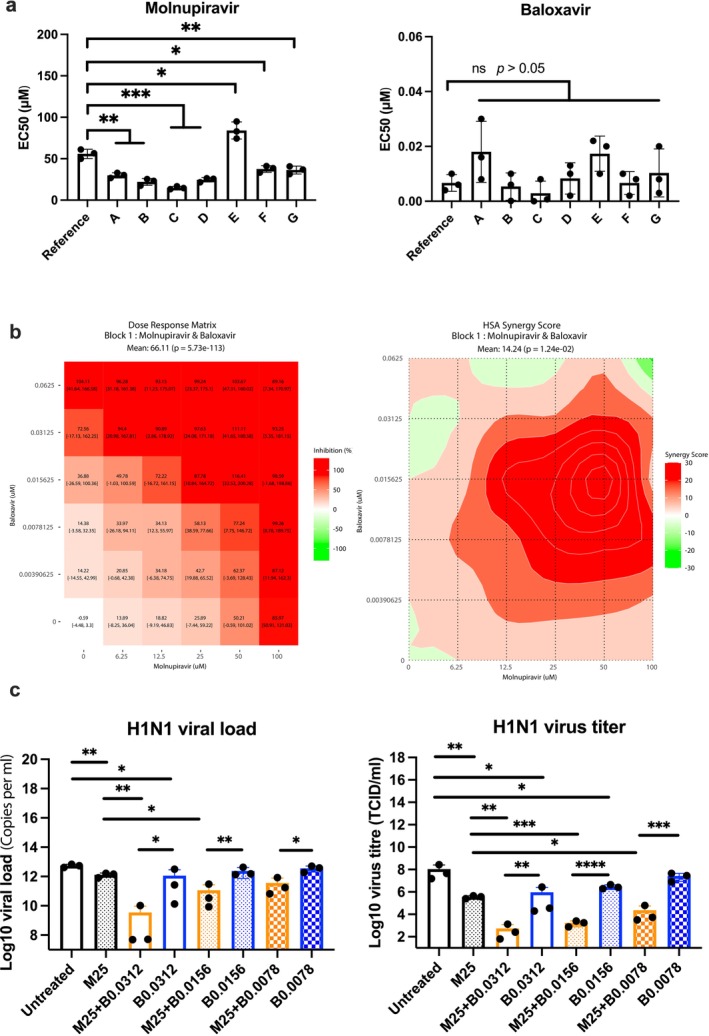
Efficacy of molnupiravir and baloxavir against influenza H1N1 strains in vitro. (a) Seven strains were selected from a total of 41 seasonal strains that showed high viral activity during oseltamivir treatment. The EC_50_ was compared to the reference H1N1 (A/Hong Kong/415742/09). (b) The synergistic effect was analyzed using Synergy Finder, and the results were based on the cell viability assay. (c) The drug concentration unit was μM. M and B refer to molnupiravir and baloxavir, respectively. The results were obtained from three independent experiments.

The 7 strains with oseltamivir EC_50_ over 800 μM were initially tested; the EC_50_ of molnupiravir and baloxavir was shown in Figure 2a. In molnupiravir treatment, 6 strains showed a lower EC_50_ compared to the 2009 reference strain, but strain E significantly increased the EC_50_ (78.64 μM). Baloxavir treatment showed no significant differences among the strains. Most strains did not show a significant decrease in susceptibility, so only the reference strain was further tested in the combination treatment. The results demonstrated that the monotherapies could significantly inhibit the viral replication (Figure 2c), and the combination therapy could protect cell viability due to synergistic effects (Figure 2b) and significantly inhibit viral replication compared to monotherapies (Figure 2c).

### 3.3 Molnupiravir Monotherapy Against the Influenza H1N1 Virus in Mice

**FIGURE 3:**
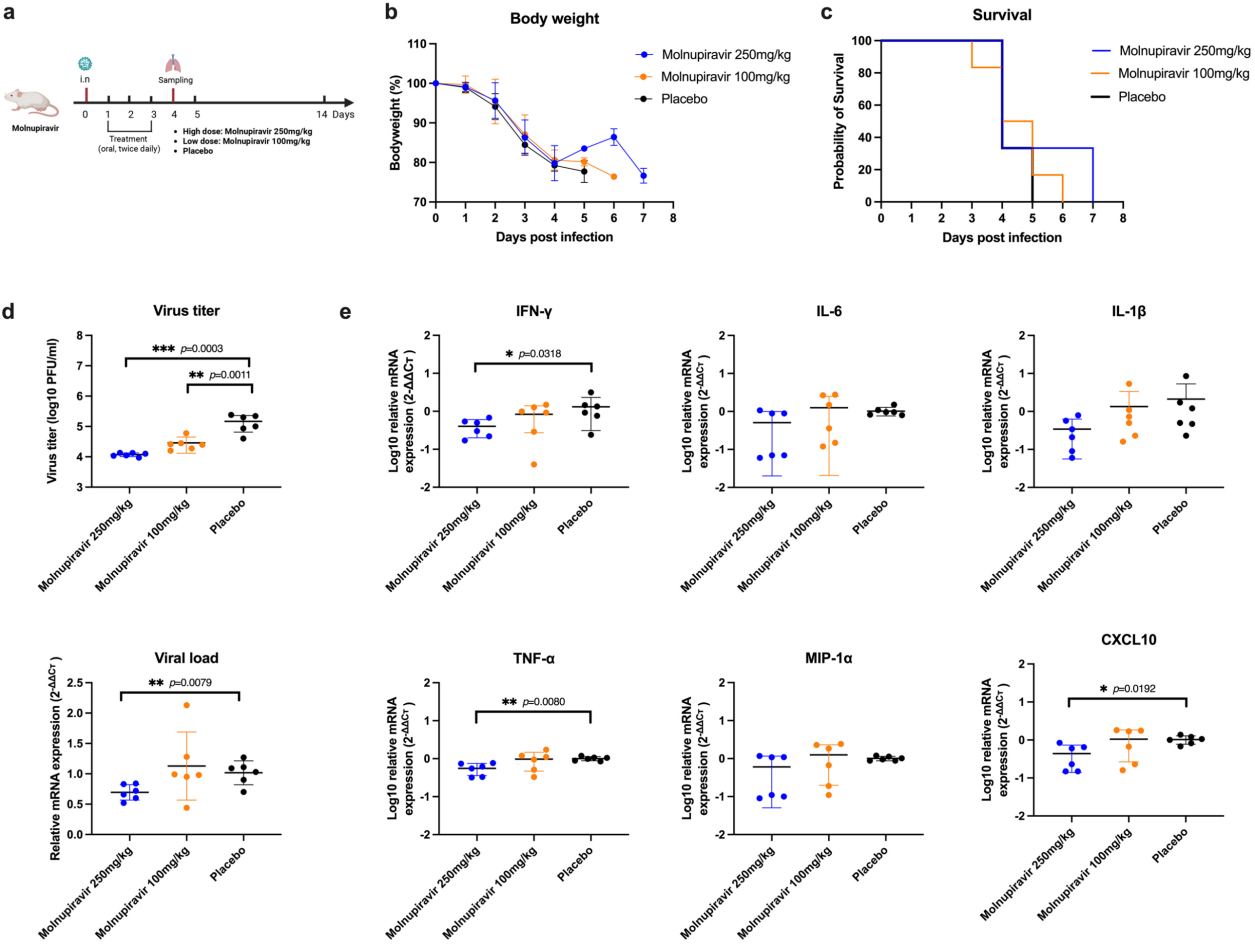
Molnupiravir treatment against the influenza H1N1 strain in vivo. (a) The procedure for animal treatments, as shown in the figure, was created with BioRender.com. (b, c) Changes in body weight and survival rates were observed over 14 days for each group (*n* = 6). (d) Virus replication in the mouse lungs at 4 dpi was measured (*n* = 6). The viral titer was determined using a plaque assay, while mRNA expression was assessed by RT‐qPCR targeting the influenza H1N1 M gene. (e) Gene expression was measured by RT‐qPCR. The results were shown as means ± SD.

**FIGURE 4:**
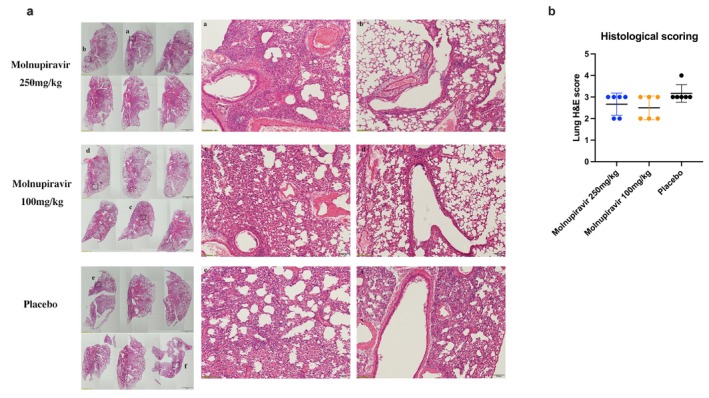
Effects of molnupiravir treatment on pathological changes after influenza H1N1 infection. (a) Lung tissue harvested at 4 dpi was stained with H&E. The images on the left showed the pathological changes in each group (*n* = 6), with a scale bar of 2 mm. The images in the middle and right (a–f) were magnified from the corresponding squares on the left, with a scale bar of 100 μm. (b) Histological scoring based on H&E staining.

Molnupiravir monotherapy did not result in significant improvements in weight loss or reductions in mortality (Figure 3b,c). Viral titers and related RNA expression were measured in the lungs of the mice at 4 dpi, revealing that virus replication decreased significantly with the high‐dose treatment (Figure 3d). The levels of IFN‐γ, TNF‐α, and CXCL10 also showed a decreasing trend in the high‐dose group (*p* < 0.05) (Figure 3e). The pathological results demonstrated that areas of inflammation and damage in the molnupiravir treatments were milder than those in the placebo group (*p* > 0.05) (Figure 4).

### 3.4 Molnupiravir and BXM Combination Treatment Against the Influenza H1N1 Virus in Mice

**FIGURE 5:**
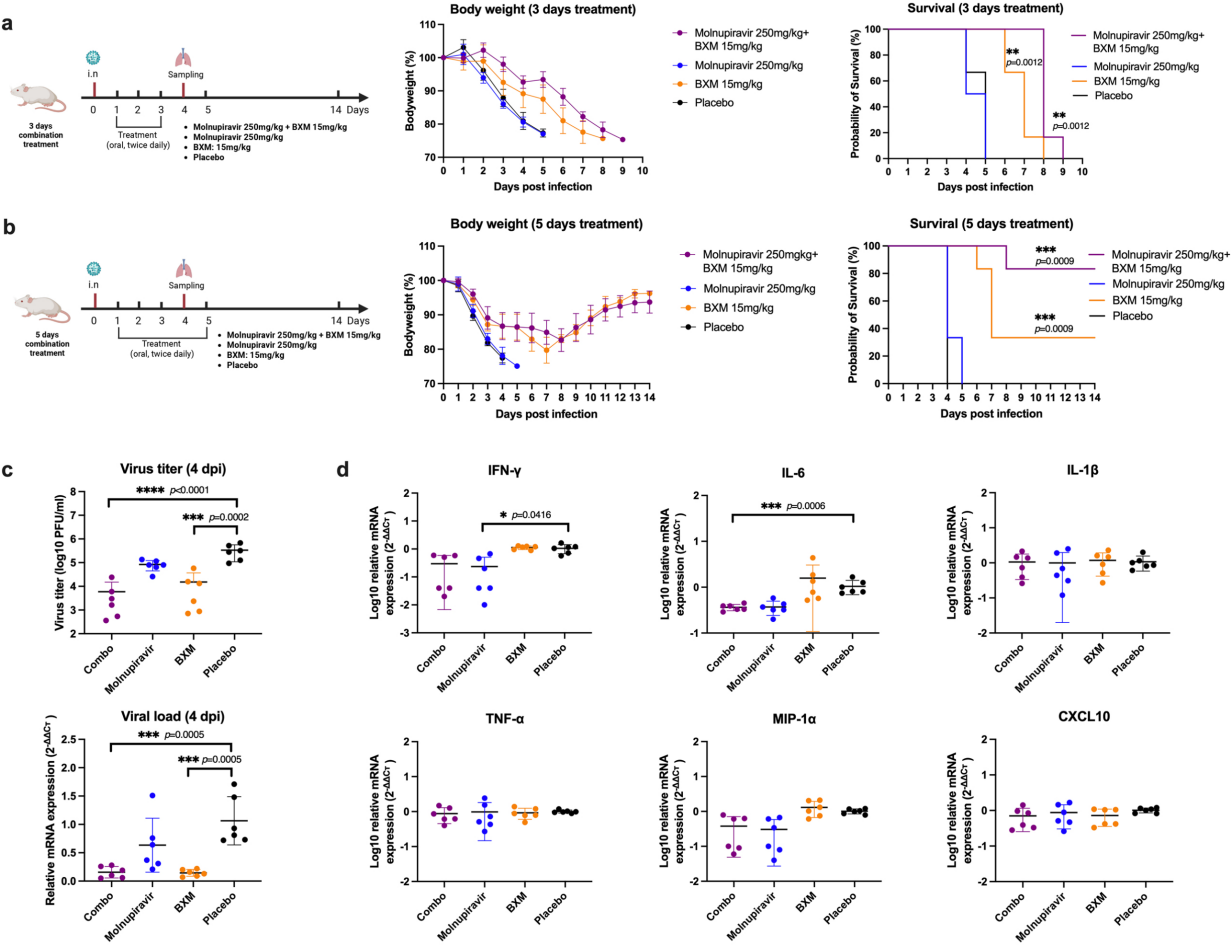
Molnupiravir and baloxavir marboxil (BXM) combination treatment against the influenza H1N1 strain in vivo. (a) Three‐day treatment started from 1 dpi, body weight changes, and 14‐day survival rates in each group (*n* = 6). (b) Five days of treatment, commencing at 1 dpi, body weight changes, and a 14‐day survival rate in each group (*n* = 6). The survival rates in each treatment group were compared with the placebo group. The figures of the experimental procedure were created with BioRender.com. (c) Virus replication in mouse lungs at 4 dpi. Virus titer was determined by plaque assay, while mRNA expression was measured by RT‐qPCR targeting the influenza H1N1 M gene. (d) Gene expression was measured by RT‐qPCR. The results were shown as means ± SD.

The treated mice exhibited slower body weight loss among those receiving a 3‐day post‐infection treatment within the BXM and combination groups. However, survival did not improve compared to the molnupiravir and placebo groups (Figure 5a). When the treatment time was extended to 5‐day post‐infection, both the BXM and combination treatments improved body weight and survival. The combination group also showed the highest survival rate (Figure 5b).

**FIGURE 6:**
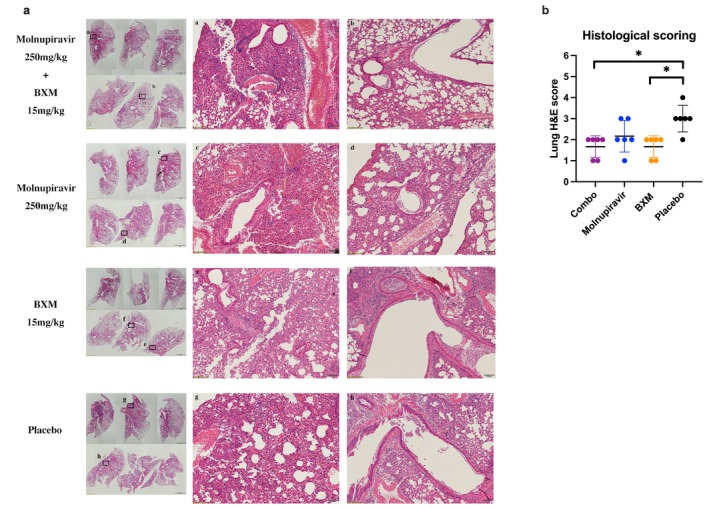
Effects of the molnupiravir and baloxavir marboxil (BXM) combination treatment on the pathological changes following influenza H1N1 infection. (a) Lung tissue harvested at 4 dpi was stained with H&E. The images on the left showed the pathological changes in each group (*n* = 6), with a scale bar of 2 mm. The images in the middle and right (a–h) were magnified from the corresponding squares on the left, with a scale bar of 100 μm. (b) Histological scoring based on H&E staining.

Virus replication and cytokine expression in the lungs were determined at 4 dpi because most mice in the placebo group had succumbed by this time due to the peak of their viral replication. The results demonstrated that the BXM and combination groups effectively reduced the viral titer and viral load (*p* < 0.05). The viral titer in the mono‐molnupiravir group showed a decreasing trend compared to the placebo group (Figure 5c). In terms of inflammatory expression, the combination and mono‐molnupiravir treatments inhibited the inflammatory response, including IFN‐γ and IL‐6 (*p* < 0.05), but BXM treatment showed no difference with the placebo treatment (Figure 5d). Histological results indicated that lung damage and inflammation in the BXM and combination groups were less severe than those in the other groups (*p* < 0.05) (Figure 6).

## 4 Discussion

Drug resistance in seasonal influenza has been a key focus of surveillance during each outbreak season. In our study, we first isolated 41 influenza H1N1 strains collected in March 2023 and determined the antiviral effect of oseltamivir in vitro. We found that the efficacy of oseltamivir dramatically decreased in some of the seasonal strains. The EC_50_ (cell viability assay) for more than half of the seasonal strains was 100‐fold higher than that of the 2009 H1N1 strain. Nevertheless, the NA inhibition assay results showed that oseltamivir did not effectively reduce the inhibitory effect on NA, thus ruling out major NA mutations leading to drug resistance.

The mechanism of NAIs prevents the cleavage of sialic acid (SA) by glycoproteins and inhibits the release of new virions [22]. In our study, the protective effect of oseltamivir on cell activity and its inhibitory impact on NA produced discrete results, leading us to speculate that mutations might occur at other positions within the virus that indirectly alter the activity of NA. Some studies have shown that altering SA‐binding properties might affect the ability of HA to bind to SA receptors, which in turn affects the NA activity [23, 24]. A synergistic effect between HA and NA functions or an increase in HA activity may enhance NA activity [23, 25]. In our study, the different results from the cell viability assay and NA inhibition assay can be explained by the experimental procedure. In the cell viability assay, the viruses were diluted to the same level based on the virus titer, allowing the assay to test both HA and NA activities simultaneously. However, the viruses were diluted according to the NA titer in the NA inhibition assay, focusing directly on NA activity and mutation while giving less consideration to indirect effects on other positions or interactions with the virus. Meanwhile, due to the differences in HA and NA activity among various strains, when comparing the correlation between HA/NA and EC_50_ in Figure 1c,d, the HA/NA results were adjusted to a uniform virus titer before performing correlation analysis. This adjustment reflects the differences in HA/NA between seasonal strains and the impact of overall changes in HA/NA activity on the effectiveness of oseltamivir. Therefore, a cell viability assay was preferred to indicate the overall impact of oseltamivir.

In the gene sequencing results, the seasonal influenza strains showed the HA having N111D mutation in comparison to the H1N1 A/Sydney/5/2021, along with more mutations relative to A/Guangdong‐Maonan/SWL1536/2019 and A/California/07/2009. These mutations were reported to be associated with a shift in host specificity, increased HA binding to SA receptors, and enhanced HA‐mediated membrane fusion [18, 19, 20, 21]. The HA mutations observed in the results may indirectly increase NA activity. The NA sequencing results also showed that strain #4 (Table 1) had a K331R mutation, which has been reported to cause mild drug resistance compared to A/Sydney/5/2021/H1N1 [16]. All 7 seasonal influenza strains exhibited mutations related to mild drug resistance compared to A/California/07/2009 [16, 17, 26]. Therefore, the virus has exhibited increasing changes in the activities of HA and NA over the years. Consequently, the effective concentration of the drug has risen gradually and may exceed the human dose limit in the future.

The efficacy of molnupiravir and baloxavir against H1N1 strains with high oseltamivir resistance was also tested; the results showed no significant resistance. The antiviral effect of molnupiravir was shown to act on RNA‐dependent RNA polymerase (RdRp), and baloxavir was indicated to inhibit vRNA synthesis [7, 27]. The efficacy of the two drugs was not significantly reduced in our highly active influenza strains. It is speculated that changes in viral activity do not primarily involve these targets. Molnupiravir inhibits virus replication by incorporating nucleoside analogs into viral RNA [28], but it may be less effective than influenza‐specific drugs. Our results confirmed that the efficacy of a BXM treatment was higher than that of molnupiravir. Combining the two drugs with different mechanisms may enhance the antiviral effect and even produce synergistic effects. Some studies demonstrated that the baloxavir/oseltamivir combination could enhance the antiviral effect, while oseltamivir monotherapy was inefficient [14, 29]. Therefore, we may consider combining baloxavir with other types of antiviral drugs, especially to reduce the impact of drug resistance caused by seasonal influenza. In our study, the molnupiravir/baloxavir combination therapy showed a synergistic antiviral effect in vitro. This finding suggested that molnupiravir may be considered if NA inhibitors are less effective or resistance emerges.

We determined the effects of molnupiravir monotherapy and molnupiravir/BXM combination therapy for influenza infection in vivo. The results showed that monotherapy did not improve the survival rate. The findings demonstrated that a mono‐molnupiravir treatment was less effective for severe infection. The effect of 3‐day and 5‐day combination treatments was tested. In the 3‐day treatment, both combination and mono‐BXM therapies prolonged the survival of mice, but they still died after 7 dpi. However, especially in the combined treatment group, the 5‐day treatment significantly improved body weight loss and the survival rate. In comparing the results of the two treatments, we believe that the 3‐day treatment may not reach plasma concentrations comparable to or sufficient for the human treatment dose [14]. Furthermore, inflammatory responses and lung damage in the late stages of infection still require ongoing treatment. Our in vivo results did not show synergistic effects compared to in vitro results. This may be attributed to molnupiravir not showing apparent positive effects compared to BXM monotherapy in severe infection. However, molnupiravir demonstrated a more effective anti‐inflammatory effect in the study on mice. This higher anti‐inflammatory effect reduced the mortality of combination therapy. We did not establish an oseltamivir treatment group in the animal study because we used the susceptible mouse‐adapted H1N1 strain, and the efficacy of NA inhibitors like zanamivir has been confirmed in the same strain [30]. Oseltamivir also showed a significant inhibitory effect in other previous studies [14, 31]. As BXM has been approved for clinical use in more countries, its drug resistance has also become a concern. The resistance is associated with substitutions of the conserved isoleucine at PA position 38, like PA‐I38T [32]. Therefore, molnupiravir and combination treatments were considered to have potential antiviral value in preventing the emergence of BXM resistance. However, the demonstrations in our study were limited by the lack of mouse‐adapted NA and PA‐resistant strains.

Our study has certain limitations. The reduction in the efficacy of oseltamivir against some recent seasonal H1N1 strains was attributed to increased NA and HA activities. However, the reasons for the increased HA and NA activities remain unclear due to the lack of information on the underlying mechanisms. The activity of seasonal influenza and the efficacy of oseltamivir treatment were not verified in animal studies due to the inconsistent sensitivity of each seasonal strain in mice. Therefore, directly comparing infectivity and therapeutic effects is challenging.

In summary, the HA and NA activities of the 2023 seasonal influenza have increased, thereby diminishing the antiviral effect of oseltamivir. Molnupiravir has shown certain anti‐influenza effects, and its combination with baloxavir can more effectively inhibit viral replication and reduce mortality in mouse studies.

## Author Contributions

**Danlei Liu:** data curation, formal analysis, conceptualization, methodology, writing – original draft, software. **Ka‐Yi Leung:** methodology, validation. **Ruiqi Zhang:** methodology, validation. **Hoi‐Yan Lam:** methodology, validation. **Yujing Fan:** methodology, validation. **Xiaochun Xie:** methodology, validation. **Wan‐Mui Chan:** methodology, validation, supervision, writing – review and editing. **Kelvin Kai‐Wang To:** writing – review and editing, supervision. **Kwok‐Hung Chan:** methodology, validation, supervision, writing – review and editing. **Ivan Fan‐Ngai Hung:** supervision, funding acquisition, writing – review and editing, project administration.

## Ethics Statement

The research was approved by the Committee on the Use of Live Animals in Teaching and Research at the University of Hong Kong (CULATR No. 24‐171) and the Institutional Review Board of the University of Hong Kong/Hospital Authority Hong Kong West Cluster (UW 13‐372 version 20210517, 31 May 2021). No written informed consent was required since only leftover specimens were used.

## Conflicts of Interest

The authors declare no conflicts of interest.

## Peer Review

The peer review history for this article is available at https://www.webofscience.com/api/gateway/wos/peer‐review/10.1111/irv.70176.

## Supporting information


**Table S1:** List of primer sequence.
**Table S2:** Cytotoxic concentration (CC) of drugs.

## Data Availability

The data supporting the study's findings are available from the corresponding author upon reasonable request.
