# Supplementary material for: Efficacy of Oseltamivir Against Seasonal Influenza H1N1 and the Efficacy of a Novel Combination Treatment In Vitro and In Vivo in Mouse Studies
Source: Influenza Other Respir Viruses. 2025 Oct 20;19(10):e70176. doi: 10.1111/irv.70176 (PMC12537536; doi:10.1111/irv.70176)
Supplement: Supplementary file 1 — Table S1: List of primer sequence. Table S2: Cytotoxic concentration (CC) of drugs. [file IRV-19-e70176-s001.docx]

**Supplementary Table 1. List of primer sequence**

| **Gene name** | **Forward primer (5’ to 3’)** | **Reverse Primer (5’ to 3’)** |
| --- | --- | --- |
| *FluA M* | GACCRATCCTGTCACCTCTGAC | AGGGCATTYTGGACAAAKCGTCTA |
|  | **Probe**: Fam-TGCAGTCCTCGCTCACTGGGCACG-BHQ1 | |
| *FluA N1* | AGACCTTGCTTCTGGGTTGA | ACCGTCTGGCCAAGACCA |
|  | **Probe**: Fam-ATCTGGACTAGCGGGAGCAGCAT-BHQ-1 | |
| *FluA H1* | GCATTTGGGTAAATGTAACAT | AGGTGTTTCCACAATGTAGG |
|  | **Probe**: FAM-CCATGAGCTTGCTGTGGAGAGTGA-BHQ1 | |
| *β-actin* | ACGGCCAGGTCATCACTATTG | CAAGAAGGAAGGCTGGAAAAG |
| *IL-1β* | GCCTTGGGCCTCAAAGGAAAGAATC | GGAAGACACAGATTCCATGGTGAAG |
| *IL-6* | TGGAGTCACAGAAGGAGTGGCTAAG | TCTGACCACAGTGAGGAATGTCCAC |
| *TNF-α* | GGTGCCTATGTCTCAGCCTCTT | GCCATAGAACTGATGAGAGGGAG |
| *IFN-γ* | AAGCGTCATTGAATCACACC | CGAATCAGCAGCGACTCCTT |
| *MIP-1α* | CCCAGCCAGGTGTCATTTTCC | GCATTCAGTTCCAGGTCAGTG |
| *CXCL10* | ATCATCCCTGCGAGCCTATCCT | GACCTTTTTTGGCTAAACGCTTTC |

**Supplementary Table 2. Cytotoxic concentration (CC) of drugs**

| Drug | CC_50_ | CC_20_ |
| --- | --- | --- |
| Molnupiravir | 788.79 ± 253.33 μM | 218.54 ± 71.33μM |
| Baloxavir | 15.49 ± 4.88 μM | 6.24 ± 2.96μM |
| Oseltamivir | >2000 μM | >2000 μM |
| DMSO (drug solvent) | >2% | >2% |

CC_50_ and CC_20_ were calculated based on cell viability. Cell viability was calculated as OD (treated - blank) divided by OD (cell control - blank). The results were obtained from three independent experiments. The results were shown as means ± SD.
